# Pulmonary Tumor Thrombotic Microangiopathy Suspected to be COVID-19 Vaccine-Related Myocarditis: A Case Report

**DOI:** 10.7759/cureus.56803

**Published:** 2024-03-24

**Authors:** Soichiro Kageyama, Takeki Ohashi, Akinori Kojima

**Affiliations:** 1 Cardiovascular Surgery, Nagoya Tokushukai General Hospital, Kasugai, JPN

**Keywords:** coronavirus vaccine-associated myocarditis, venoarterial extracorporeal membrane oxygenation, circulatory failure, pulmonary hypertension, pulmonary tumor thrombotic microangiopathy

## Abstract

Pulmonary tumor thrombotic microangiopathy (PTTM) is a very rare condition that can lead to acute severe pulmonary hypertension and circulatory failure. It is caused by tumor cell microvascular obstruction and is usually difficult to diagnose; in fact, it is often diagnosed after death. We report the case of a patient who experienced a sudden cardiac arrest and developed severe pulmonary hypertension two days after receiving the coronavirus disease (COVID-19) vaccine. The patient was initially diagnosed with vaccine-associated myocarditis, and venoarterial extracorporeal membrane oxygenation (VA-ECMO) implantation with median sternotomy was performed. The patient survived for more than two weeks. PTTM was later diagnosed during a pathological autopsy.

## Introduction

Pulmonary tumor thrombotic microangiopathy (PTTM) is a rare condition caused by tumor cell microvascular obstruction and can result in acute severe pulmonary hypertension and circulatory failure. Most cases of microscopic tumor embolisms are difficult to diagnose using imaging studies and are not recognized until the patient has died [1]. We present the case of a patient who experienced a sudden cardiac arrest and developed severe pulmonary hypertension two days after receiving a COVID-19 vaccine. The patient was initially diagnosed with coronavirus disease (COVID-19) vaccine-associated myocarditis, and extracorporeal circulation therapy was administered. An extremely rare primary cholangiocarcinoma was diagnosed during pathological autopsy.

This case report relied on existing patient information without intervention for research purposes, and the requirement for obtaining written patient consent was waived.

## Case presentation

A 61-year-old female with no relevant medical history presented to a hospital with sudden dyspnea. She had received a COVID-19 vaccine (Pfizer-BioNTech COVID-19 vaccine: BNT162b2 mRNA) two days before the onset. Transthoracic echocardiography (TTE) revealed right ventricle (RV) dilatation, left ventricle (LV) collapse, and pulmonary hypertension. Pulmonary artery embolism was initially suspected. However, contrast-enhanced CT showed no obvious contrast deficit in the pulmonary artery. Her hemodynamics deteriorated during the examination, leading to cardiac arrest. The patient was treated with venoarterial extracorporeal membrane oxygenation (VA-ECMO) via the femoral artery and vein, and her heartbeat resumed. She was transferred to our hospital for further treatment.

**Figure 1:**
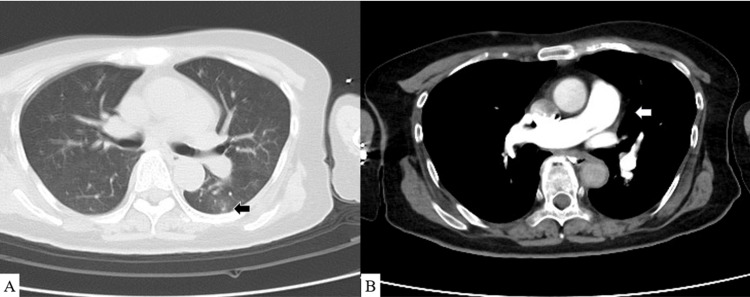
CT findings on the first day (A) Black arrow: pulmonary ground-glass opacity is observed. (B) White arrow: no contrast deficit is observed in the pulmonary artery CT: computed tomography

Blood tests at our hospital revealed an elevated inflammatory response and liver and renal dysfunction. TTE revealed LV collapse and a low ejection fraction (LVEF) of 38%. Contrast-enhanced CT revealed a pulmonary ground-glass opacity but no pulmonary artery thrombi (Figure 1).

COVID-19 vaccine-related myocarditis was suspected given sudden post-vaccine severe cardiac decompensation, and steroid pulse therapy and high-dose gamma globulin treatments were initiated. Nitric oxide therapy for pulmonary hypertension was also initiated, and an Impella® heart pump (Abiomed, Danvers, MA) was placed for LV decompensation and reduced LV afterload. This stabilized the hemodynamics (VA-ECMO flow: 1.8 L/min, Impella® flow: 2.2 L/min, blood pressure: 100/87 mmHg, pulmonary artery pressure: 43/25 mmHg, and central venous pressure: 5 mmHg). A myocardial biopsy was performed as well.

**Figure 2:**
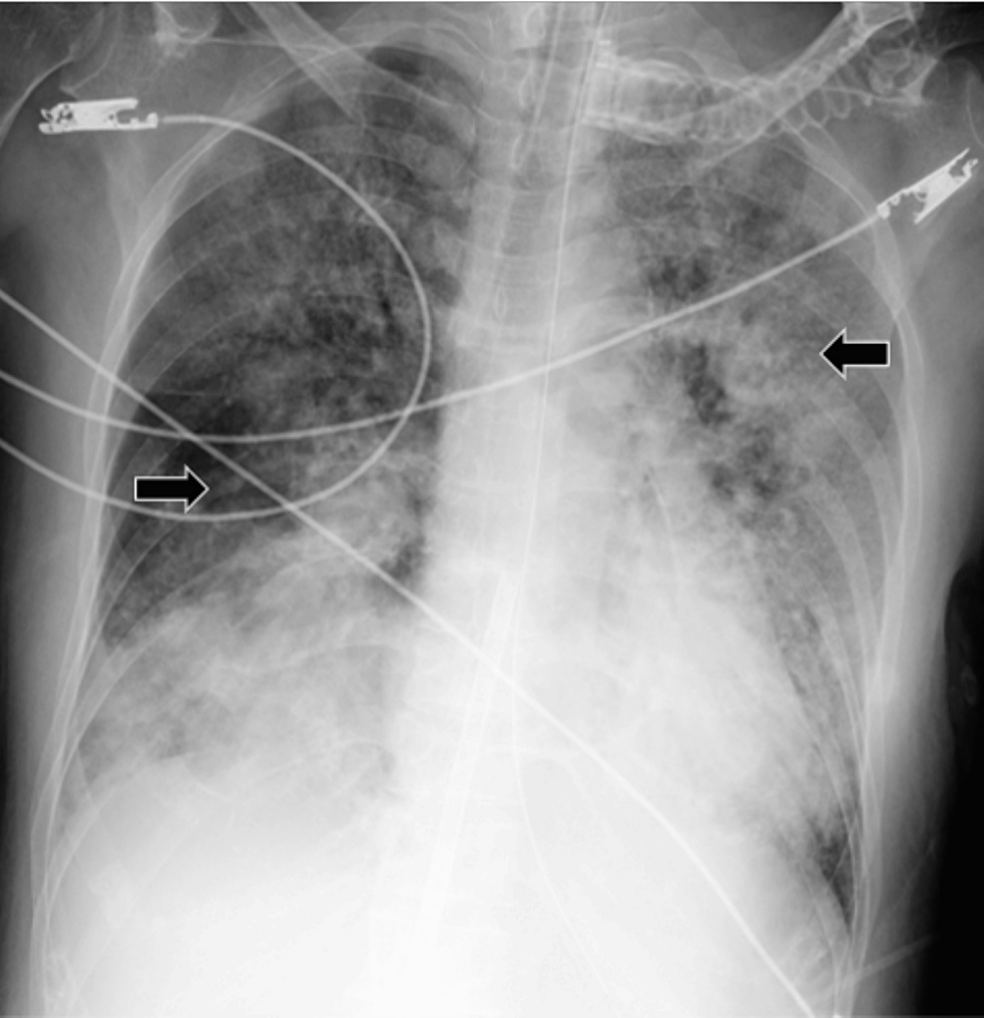
Chest radiography findings on the seventh day Black arrow: chest radiograph reveals severe pulmonary congestion

**Figure 3:**
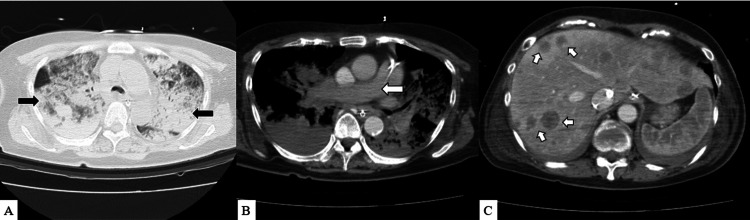
CT findings on the eighth day (A) Black arrow: severe lung field consolidation is observed. (B) White arrow: no contrast defect of the pulmonary artery is observed. (C) White arrow: multiple masses are observed in the liver CT: computed tomography

On the seventh day, the flow through the Impella® heart pump dropped and could not be maintained despite the reduction in the ECMO flow. The Impella® heart pump was removed. Transesophageal electrocardiography showed further RV enlargement and LV collapse. This seemed insufficient for blood flow from the right to the left heart system. A myocardial biopsy revealed no significant inflammatory cellular infiltrates or evidence of amyloidosis or sarcoidosis. Chest radiography revealed severe pulmonary congestion. Follow-up CT showed the worsening of the lung field consolidation, and contrast defects of the pulmonary arteries remained absent. Multiple masses in the liver were not observed on a previous CT scan (Figures 2, 3). The cardiac function improved; however, the native pulmonary hypoxia worsened (respirator FiO_2_: 100%; ECMO flow: 3.5 L/min; left radial artery PaO_2_: 47 mmHg; left radial artery PaCO_2_: 49 mmHg). Therefore, we decided to upgrade the VA-ECMO to central ECMO via the ascending aorta and femoral vein for cerebral protection.

**Figure 4:**
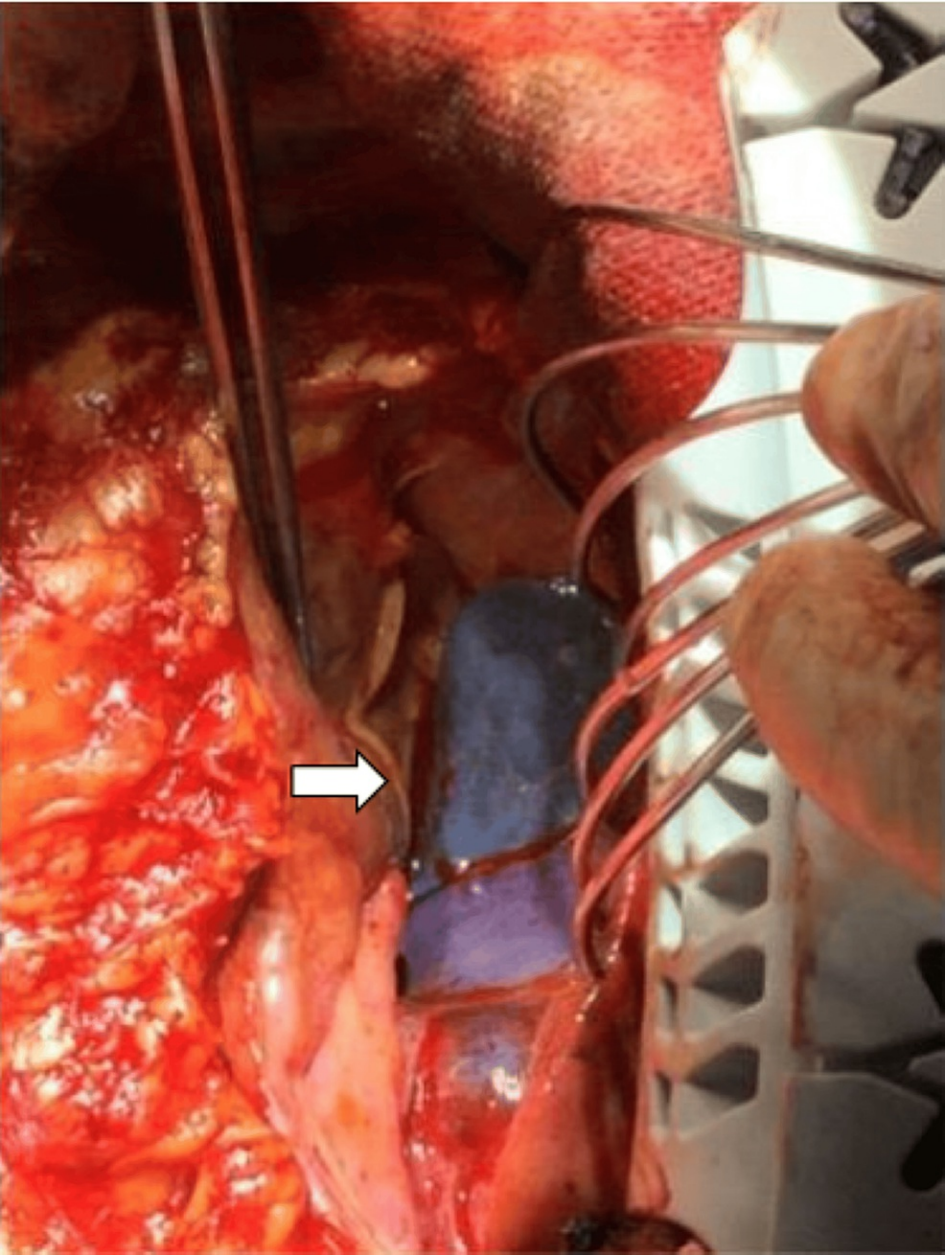
Operative findings White arrow: the right lower lobe of the lung appears dark reddish and hardened

**Figure 5:**
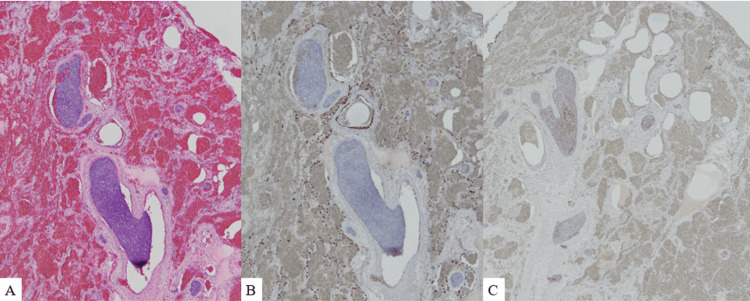
Pathological findings (A) Lung biopsy reveals tumor embolism in the peripheral pulmonary arteries. (B) Immunostaining is negative for TTF-1. (C) Immunostaining is positive for CD56

On the eighth day, central ECMO attachment with a median sternotomy was performed. Central ECMO reduced the pulmonary artery pressure (54/32 mmHg to 10/9 mmHg); the cerebral oxygenation also improved (left radial artery PaO_2_: 186 mmHg). Part of the lung was dark reddish and had hardened (Figure 4). Furthermore, a lung biopsy performed during surgery revealed tumor embolism in almost all peripheral pulmonary arteries (Figure 5). This suggested that the PTTM was due to a malignant tumor with an unknown primary site and metastasis to the liver. After that, multiple organ failure developed gradually, and the patient died on the 15th day of the disease onset. Histological examination of the tumor cells revealed a small cell carcinoma. The pulmonary artery was filled with tumor cells and thrombi, and no primary tumors were found in the lungs. Multiple 1-2 cm nodules were scattered throughout the liver, and some formed abscesses. The pathological diagnosis was of an intrahepatic cholangiocarcinoma, and the mass in the liver was considered the primary tumor with intrahepatic metastasis. Immunostaining was negative for TTF-1 and positive for CD56; positivity for TTF-1 and CD56 is often observed in primary small-cell lung carcinomas and intrahepatic cholangiocarcinomas, respectively (Figure 5).

## Discussion

Diagnosing PTTM is generally challenging; 79% of the cases are diagnosed after death [2]. Patients may present with cough (85%), dyspnea (95%), hypoxemia (95%), anemia (84%), and thrombocytopenia (95%). CT predominantly reveals pulmonary ground-glass opacities (82%) and nodular shadows (86%). The average duration from onset to death is 9.5 weeks. Advanced therapies, such as phosphodiesterase inhibitors, endothelin-receptor antagonists, prostacyclin analogs, inhaled nitric oxide, and antineoplastic agents, have been shown to prolong life by several months; however, several challenges remain [3]. Gastric adenocarcinoma (59%) is the most common primary site for PTTM; conversely, cholangiocarcinoma is extremely rare and has been limited to case reports [3,4]. Most small-cell carcinomas occur in the lungs, and primary small-cell carcinomas of the liver are extremely rare [5]. However, the association between PTTM and COVID-19 vaccines remains unclear.

Our patient was initially diagnosed with cardiogenic shock due to myocarditis and managed with VA-ECMO and the Impella® blood pump. However, blood flow from the right to left native cardiac system became more inhibited as the disease progressed, and the flow through the Impella® pump could not be maintained. These hemodynamic patterns mimicked those associated with pulmonary artery embolism, and PTTM was suspected. VA-ECMO is useful for hemodynamic patterns, such as those for pulmonary artery embolism; however, the cardiac function is preserved, and the brain and upper body are perfused with deoxygenated blood via the native circulation system in case of coexisting respiratory dysfunction [6]. In such cases, upgrading to central ECMO may be useful; herein, the oxygenated blood is sent to the ascending aorta, and the coronary artery or cerebral vessels remain oxygenated. Conversely, veno-venous ECMO, wherein oxygenated blood is sent to the pulmonary artery, may be ineffective in cases of PTTM with an LV collapse due to an inhibited blood flow from the right to the left cardiac system.

## Conclusions

PTTM is extremely rare and can be difficult to diagnose because patients experience rapidly worsening oxygenation and hemodynamics. VA-ECMO or central ECMO may be useful for maintaining circulation and oxygenation; however, these are not radical treatments for PTTM. Our patient died 15 days after the onset of this disease. PTTM should always be considered in cases of acute pulmonary hypertension of an unknown etiology.
